# Intuitive and Minimally Invasive Surgical Technique for Comminuted Mid-Shaft Clavicle Fractures: Fixation with an Anterior Mini Plate and Superior Locking Compression Plate

**DOI:** 10.3390/jcm14030999

**Published:** 2025-02-04

**Authors:** Seungwoo Ok, Seong-Meen Yoon, Sungwook Choi

**Affiliations:** Department of Orthopedic Surgery, Jeju National University Hospital, Jeju 63241, Republic of Korea; vinceok89@gmail.com (S.O.); osyoon37182@gmail.com (S.-M.Y.)

**Keywords:** locking compression mini plate, clavicle dual plating, multifragmentary clavicular fracture

## Abstract

**Background**: We have applied an anterior locking compression mini plate in addition to a conventional superior locking compression plate for the treatment of wedge or multifragmentary clavicular fractures. **Methods**: Medical and radiologic data were retrospectively reviewed for patients who underwent surgical fixation with an anterior locking compression mini plate and conventional anatomical locking compression plate in a clavicle fracture of AO/OTA 15.2 B and 15.2 C. The primary outcome was bone union, and the secondary outcome was postoperative complications associated with the procedure. The functional outcomes included the Visual Analog Scale (VAS), University of California at Los Angeles Shoulder Scale (UCLASS), and Constant Shoulder Scale (CSS). **Results**: Nineteen patients with AO/OTA 15.2 B and 2 C clavicular fractures were followed for an average of 16 months (range: 12–30). The average patient age was 41 (range: 21–76) years, and 17 male and 2 female patients were included. The most common cause of clavicle fractures was sports activity (36.8%). A total of 10 patients had AO/OTA classification 15.2 C, and 9 patients had AO/OTA classification 15.2 B clavicular fractures. Primary fracture union healing was observed in all 19 (100%) patients, and the average time to bone union was 11 weeks (range: 7~21). There was no fixation failure or postoperative infection. The mean VAS, UCLASS, and CSS scores at the final follow-up were 0.6, 33.4, and 65 on a 75-point scale (87 on a 100-point scale), respectively. **Conclusions**: Dual plating using an anterior mini plate with a superior LCP could be considered as an option to minimize soft-tissue injury in comminuted mid-shaft clavicle fractures.

## 1. Introduction

**Figure 1:**
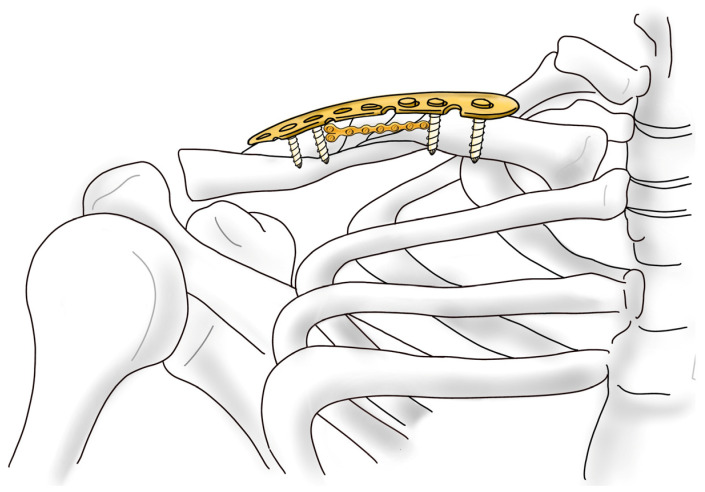
A schematic representation of surgery using anterior mini plating and superior LCP plating.

Clavicular fractures make up 2.6% of all adult fractures, and up to 80% of clavicular fractures are localized in the mid-shaft. Since the early 1990s, conservative treatment, developed by Neer, has been regarded as the treatment of choice [1]. However, a number of studies have shown that nonsurgical treatment frequently results in comminution, displacement, and shortening, which increases the risk of symptomatic malunion and nonunion [2]. Additionally, there have been reports indicating that patients with fractures who did not undergo surgery exhibit reduced shoulder strength [3]. Therefore, recent attempts have been made to perform surgical treatment on patients with clavicle fractures. Notably, open reduction and internal fixation (ORIF) using plates is commonly employed. Additionally, depending on the fracture pattern, various approaches involving open or closed reduction using intramedullary (IM) pins, wires, or nails are being attempted [4,5]. In cases where the fracture pattern is a displaced comminuted fracture, the nonunion rate has been reported to be as high as 20% when surgical treatment is not performed. As a result, most surgeons prefer to opt for surgical intervention in comminuted clavicle fractures and plate fixation is often preferred over intramedullary nailing for comminuted clavicle fractures due to its superior stability for complex fracture patterns, and the lower risk of complications like pin migration and nonunion [6,7,8,9]. However, plating has been reported to carry certain disadvantages, including the risk of damage to underlying neurovascular structures and excessive stripping at the fracture site, which can compromise the biology of bone healing and increase the risk of infection [10,11]. The authors have considered methods to reduce such complications while also enabling relatively straightforward and intuitive surgical procedures for complex comminuted fractures. Although good results were achieved using the mini-open technique for surgery, there were many cases in which reduction was challenging due to the nature of comminuted fractures, where fragments tend to move across three planes [12]. In addressing these complex fractures, the authors focused on the use of additive fixation with mini plates [13]. These plates are utilized with a purpose similar to that of lag screws but are easier to apply (Figure 1). Moreover, the stability observed during surgery was robust, leading to favorable outcomes in cases of comminuted patella fractures [14]. Based on studies demonstrating that dual plating with mini plates provides sufficient mechanical stability, by fixing the smaller fragments to the main fragment using a mini plate, the authors effectively converted a complex fracture pattern into one resembling a simpler fracture configuration [15]. Following this, the fracture surface was made more amenable to reduction, and a superior locking compression plate (LCP) was applied. To minimize periosteal damage, only the screw insertion sites were exposed through mini-open incisions, thereby facilitating fixation in a manner consistent with minimally invasive plate osteosynthesis (MIPO).

The purpose of this study is to introduce the dual-plate fixation method using the mini-open technique for comminuted clavicle fractures and to report the outcomes associated with this approach.

## 2. Materials and Method

### 2.1. Clinical Series

A total of 19 patients who received comminuted mid-shaft clavicular fracture treatment at our hospital from May 2018 to November 2020 were retrospectively reviewed. This study was approved by our Institutional Review Board. Patients aged 21–76 years were included. The inclusion criteria for this study were (a) fracture type of AO/OTA classification 15.2 B or 15.2 C, (b) internal fixation with dual plates (anterior mini plate and superior clavicle LCP), and (c) normal shoulder function before injury. The exclusion criteria included (1) proximal or distal fracture, (2) skeletal immaturity, (3) open fracture, (4) pathological fracture, or (5) neurovascular injury. The average patient age at the time of injury was 41.2 years. The most common cause of injury was sports (36.8%), followed by slip down (26.3%), fall down (21%), and traumatic accidents (15.7%). A total of 9 cases were classified as AO/OTA classification type 15.2 B, while 10 cases were 15.2 C.

All patients were initially evaluated by clavicle anteroposterior (AP) and clavicle lordotic radiographs of both clavicles. Clavicle fractures were categorized on the basis of the Orthopedic Trauma Association Classification (AO/OTA) scheme based on the initial AP plain radiograph of the clavicle, along with the 3D reconstructed computerized tomography (CT) scans performed to understand the fracture anatomy and preoperative planning.

All patients were treated with one anterior 2.0 mm LCP compact hand mini plate (DePuy-Synthes^®^. Warsaw, IN, USA) with one superior 3.5 mm LCP superior anterior clavicle plate (DePuy-Synthes^®^. Warsaw, IN, USA). All surgical procedures were performed by a single orthopedic-trauma-specialized surgeon (S.C.) at our hospital trauma center.

Operative time, postoperative complications, and clinical outcomes were assessed by reviewing admission and outpatient medical records. The time to osseous union was evaluated by periodic radiologic assessment. All patients underwent follow-up at regular postoperative intervals (1, 3, 6, and 12). Simple radiographs (clavicle AP, clavicle lordotic view) were obtained during the immediate postoperative period and at each follow-up outpatient visit. Both the immediate postoperative and the final follow-up radiographs were compared to evaluate the accuracy of reduction and final displacement.

The primary outcome measure was osseous union, defined as the loss of the fracture line and the presence of bony trabecular continuity on simple radiographs. Functional scores, including the Constant Shoulder Score (CSS), UCLA Shoulder Score, and visual analog scale (VAS) score were assessed as secondary outcomes. However, for the Constant Shoulder Score, due to the absence of equipment to assess muscle grip power (worth 25 points) at our institution, the evaluation was conducted out of a total of 75 points, excluding this item. These scores were all measured by a single specialized nurse at 1-year postoperatively.

### 2.2. Surgical Protocol

**Figure 2:**
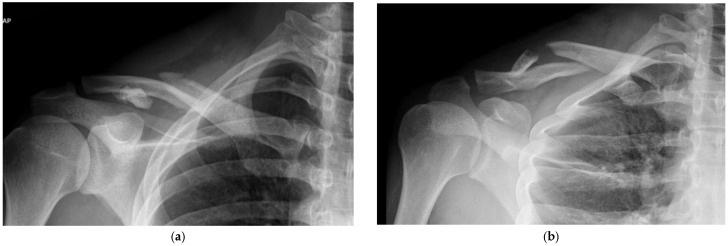
A 22-year-old male patient was diagnosed with a right clavicle mid-shaft fracture during sports activity. Fracture findings corresponding to AO/OTA classification 15.2 C with multiple fragments were confirmed on clavicle AP (**a**) and lordotic view X-rays (**b**).

**Figure 3:**
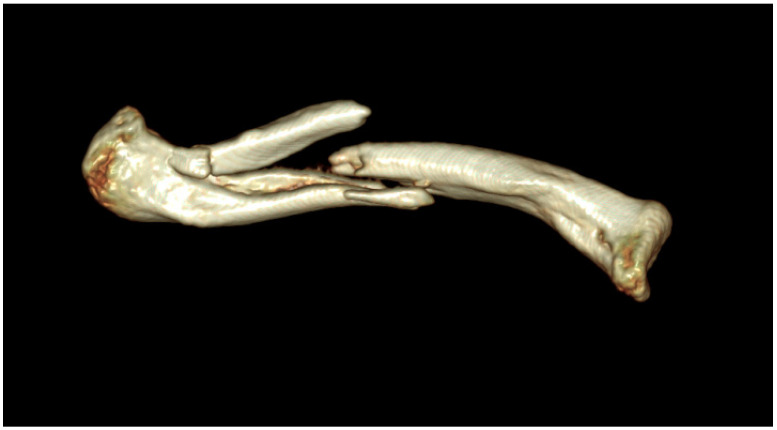
3D-reconstructed CT image of the same patient. Through this image, the pattern of the fracture can be seen more intuitively.

**Figure 4:**
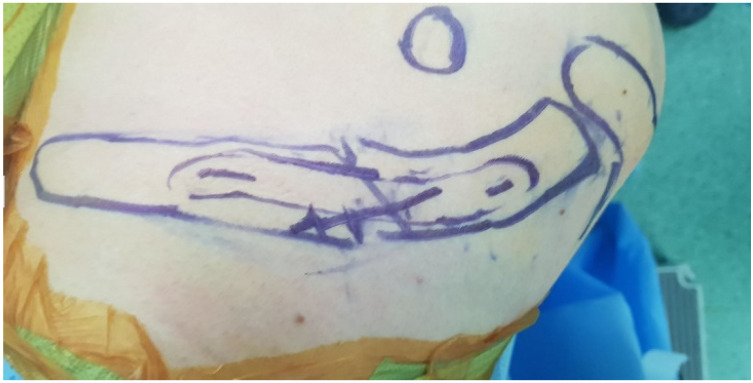
The patients were placed in the beach-chair position under general anesthesia with the head and neck tilted away from the surgical site. After applying the surgical drape, the overall contour of the clavicle and the fracture site were marked using a surgical marker, guided by C-arm imaging.

**Figure 5:**
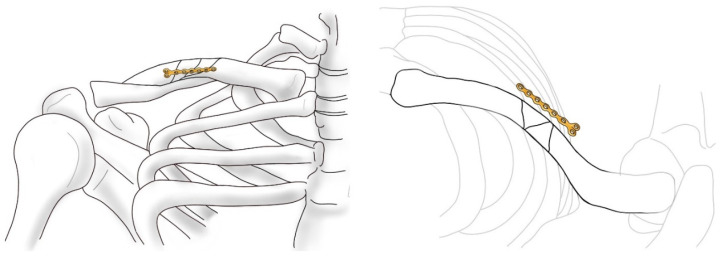
A small transverse incision, approximately the size of the fracture site, was made along the clavicle fracture line. Typically, this incision measured around 5 cm in length. The reduction was performed with one mini plate (2.0 mm LCP compact hand, DePuy-Synthes^®^).

**Figure 6:**
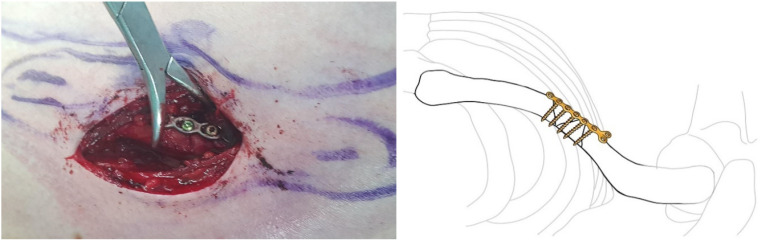
By fixing wedge or comminuted fragments to the main fragment using mini plate, fractures are converted to simpler fractures to apply superior LCP. The length of the mini plate only needs to be sufficient to cover the length of the fracture site.

**Figure 7:**
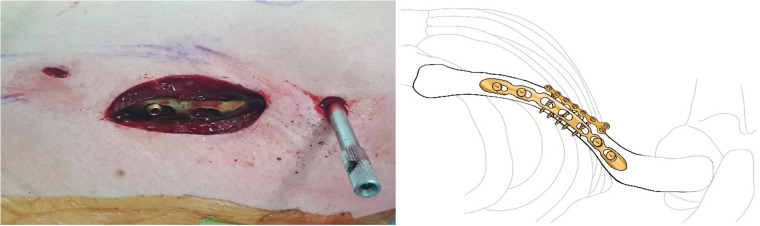
The clavicle LCP (3.5 mm LCP superior clavicle system, DePuy-Synthes^®^) is applied and fixed to the superior side of the clavicle, employing a technique similar to minimally invasive plate osteosynthesis (MIPO). To fix the plate, screws must be inserted in a compression mode using the eccentric position of the screw holes in the plate. Since the mini plate provides additional stability, it is not strictly necessary to secure both ends of the superior plate with three screws each.

The fracture pattern is preoperatively assessed using X-ray and 3D reconstructed CT images (Figure 2 and Figure 3). The patients were placed in the beach-chair position under general anesthesia with the head and neck tilted away from the surgical site. After applying the surgical drape, the overall contour of the clavicle and the fracture site were marked using a surgical marker, guided by C-arm imaging (Figure 4). A small transverse incision, approximately the size of the fracture site, was made along the clavicle fracture line. Typically, this incision measured around 5 cm in length. The reduction was performed with one mini plate (2.0 mm LCP compact hand, DePuy-Synthes^®^) (Figure 5). By fixing wedge or comminuted fragments to the main fragment using a mini plate, fractures are converted to simpler fractures to apply a superior LCP. The length of the mini plate only needs to be sufficient to cover the length of the fracture site (Figure 6). In the 19 cases studied, the maximum number of fragments, except two main fragments, was three, and a single mini plate was adequate for fixation. Following this process, a clavicle LCP (3.5 mm LCP superior clavicle system, DePuy-Synthes^®^) was applied and fixed to the superior side of the clavicle, employing a technique similar to minimally invasive plate osteosynthesis (MIPO) (Figure 7). To fix the plate, screws must be inserted in a compression mode using the eccentric position of the screw holes in the plate. This technique ensures that compression force is applied between fragment A (the main fragment connected to small fragments with the mini plate) and fragment B (the opposing main fragment). Although skin discomfort due to the implant can be equally pronounced with superior plating compared to anterior plating [16], the decision to use a superior plate with an anterior mini plate was based on studies indicating that superior plating offers greater mechanical stability [17]. Subsequently, the degree of fracture reduction and the screw length were checked using a C-arm fluoroscopy machine. After verifying these, the surgical site was irrigated, a silastic drain was inserted, and the incision was closed with layer-by-layer suturing to complete the procedure.

**Figure 8:**
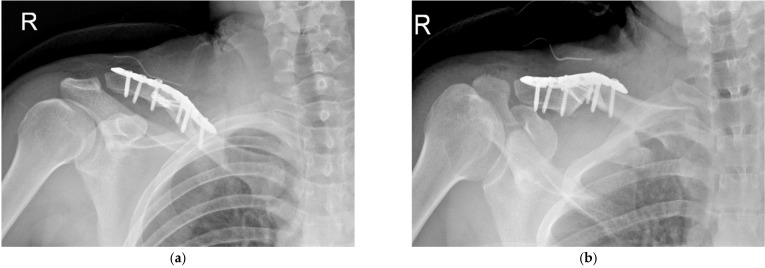
Postoperative right clavicle AP (**a**) and lordotic (**b**) X-rays.

**Figure 9:**
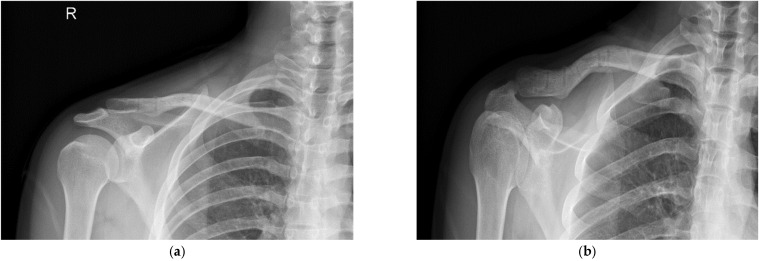
Right clavicle AP (**a**) and lordotic (**b**) X–rays, which were taken 2 months after implant removal and 14 months after first operation.

Patients were given a sling for comfort for the initial 7 days after the procedure, followed by the introduction of range-of-motion exercises. Eight weeks post-surgery, once evidence of bone union was confirmed on the X-ray and clinical assessment showed minimal pain, muscle strengthening exercises were initiated. Simple radiographs (clavicle AP, clavicle lordotic view) were obtained during the immediate postoperative period and at each follow-up outpatient visit (Figure 8 and Figure 9).

## 3. Results

A total of 19 patients with AO/OTA 15.2 B and 15.2 C clavicle fractures were followed up for at least 12 months (range: 12–25). All fractures were treated with one anterior mini plate (2.0 mm compact hand, DePuy-Synthes^®^) and with one superior clavicular LCP (3.5 mm LCP superior clavicle plate, DePuy-Synthes^®^). The mean operation time was 107 min (range 79–154).

Primary fracture union healing was observed in all 19 patients. The average time to bone union was 11 weeks (range 7–21) with no infection, malalignment, implant failure, shortening, or nonunion. In one case, the screw of the superior plate was judged to be too deep to damage the pleura on postoperative x-ray (although the C-arm fluoroscopy performed during surgery appeared satisfactory), so screw repositioning was performed on the day of surgery.

For the Constant Shoulder Score (CSS) at the 1-year follow-up, the average score was 65 (range 41–73) based on a 75-point scale (87 on a 100-point scale), excluding the muscle grip power assessment.; for the UCLA Shoulder Score, the average value obtained at the 1-year follow-up was 33.4 (range 22–35); and for the VAS, it was 0.6 (range 0–4) (Table 1).

Following surgery, one patient reported numbness around the surgical site, with symptoms persisting for six months before improving. Another patient experienced symptoms for more than six months postoperatively. Detailed history taking revealed that the symptoms had been present even prior to the injury and physical examination revealed generalized paresthesia extending from the surgical site to the upper arm. EMG/NCS testing confirmed C4-5 radiculopathy.

## 4. Discussion

Historically, based on studies by Neer and others, mid-shaft clavicle fractures were considered to yield sufficiently satisfactory outcomes with conservative treatment, and this approach was commonly practiced [1]. However, recent research has reported that conservative treatment is associated with an increased rate of nonunion, and this trend has also been observed in cases of comminuted fractures [3,18].

In cases of comminuted mid-shaft clavicle fractures, we considered a surgical technique that is relatively straightforward, minimizes injury to the soft tissue, and provides sufficient fixation stability to promote bone healing. Based on studies indicating that mini plates can provide sufficient stability [15], it was believed that performing a mini-open procedure on the anterior side and achieving partial fixation with a mini plate would minimize soft-tissue injury. To maintain this concept, the superior LCP was applied in a manner similar to MIPO. While the role of the mini plate could be substituted with a lag screw technique, based on the author’s experience, in cases of more severe comminution, using a mini plate was technically easier than utilizing a lag screw.

Since the purpose of this study was to introduce a surgical technique, it has several clinical limitations. The first major limitation is the small sample size of 19 cases, which is insufficient for robust analysis. Additionally, the absence of a control group is a significant limitation, hindering the ability to make accurate comparative evaluations. A comparison of union rates, complication rates, and clinical scores with a control group should be conducted to further evaluate the effectiveness of the surgical technique. While we describe this surgical method as straightforward, proving this claim would require future controlled studies. Such studies should compare the overall surgical time, as well as the time needed for reduction and fixation (including the application of the anterior mini plate and superior LCP), according to the type of fracture and the number of fragments. This comparison would provide more concrete evidence supporting the intuitiveness of the technique.

Although not extensively discussed in this study, irritation caused by the superior LCP is a common issue. Among the 19 cases, 5 patients reported such irritation. As mentioned earlier, the decision to use the superior LCP in this study was based on research indicating its superior mechanical stability compared to the anterior plate [17]. However, if it provides sufficient mechanical stability to support bone healing, the anterior LCP and superior mini plate could also be considered as alternative options.

Finally, in this study, one patient out of the 19 cases reported numbness. It has been reported that the occurrence of numbness is lower with the MIPO technique compared to the traditional open reduction and internal fixation technique [19]. Although the surgical technique used in this study was not MIPO, the advantages of the mini-open technique, which minimizes soft-tissue stripping, may be reflected in these findings.

## 5. Conclusions

In comminuted mid-shaft clavicle fractures, this surgical technique—performing a mini-open procedure at the fracture site, followed by partial reduction and fixation using a mini plate on the anterior side and applying a clavicle LCP on the superior side in a manner similar to MIPO—resulted in primary bone healing in all 19 cases without complications. Additionally, at one year postoperatively, the evaluated Constant Shoulder Score, UCLA Shoulder Score, and VAS indicated favorable outcomes. However, this study has significant limitations, including a small sample size of 19 cases and the absence of a control group, which precludes precise comparative evaluation. Further research is necessary to address these limitations.

## Figures and Tables

**Table 1:** 1-year follow-up results from clavicular fractures treated with dual plating.

Case	Sex	Age	Injury	AO Classification (15.2)	Plate	Time to Union (Weeks)	Complications	Shortening (mm)	VAS	CSS	UCLASS
1	M	30	sports	B	Dual	12	None	0	0	52	35
2	M	36	TA	C	Dual	21	None	0	0	73	33
3	M	21	SD	C	Dual	9	None	0	0	53	35
4	M	22	Sports	B	Dual	7	None	0	0	69	35
5	M	43	Sports	B	Dual	13	None	0	0	71	33
6	F	57	FD	B	Dual	11	None	0	0	68	33
7	M	35	FD	C	Dual	11	None	0	0	66	33
8	M	55	SD	B	Dual	12	None	0	0	64	31
9	M	58	TA	C	Dual	11	None	2	0	41	22
10	M	25	Sports	C	Dual	15	None	3	4	73	35
11	M	51	FD	C	Dual	11	None	7	0	72	35
12	F	39	SD	B	Dual	14	None	0	3	63	35
13	M	28	Sports	B	Dual	10	None	0	0	62	31
14	M	35	Sports	B	Dual	14	None	3	0	58	35
15	M	40	Sports	B	Dual	10	None	3	1	71	35
16	M	35	SD	C	Dual	10	None	0	0	72	35
17	M	76	FD	C	Dual	10	None	0	0	71	35
18	M	34	TA	C	Dual	15	None	0	0	68	33
19	M	64	SD	C	Dual	9	None	0	0	69	35
mean		41.2				11		0.95	0.6	65	33.4

M: male, F: female, TA: traffic accident, SD: slip down, FD: fall down, VAS: visual analog scale, CSS: Constant Shoulder Score, UCLA: University of California Los Angeles Shoulder Score. Complications: nonunion, malunion, infection, implant failure, shortening.

## Data Availability

The dataset used and/or analyzed during the current study available from the corresponding author on reasonable request.
